# Good to Know: Baseline Data on Feed Intake, Fecal Pellet Output and Intestinal Transit Time in Guinea Pig as a Frequently Used Model in Gastrointestinal Research

**DOI:** 10.3390/ani11061593

**Published:** 2021-05-28

**Authors:** Kristin Elfers, Yvonne Armbrecht, Gemma Mazzuoli-Weber

**Affiliations:** Institute for Physiology and Cell Biology, University of Veterinary Medicine Hannover, Foundation, 30173 Hannover, Germany; yvonne.armbrecht@tiho-hannover.de (Y.A.); Gemma.Mazzuoli-Weber@tiho-hannover.de (G.M.-W.)

**Keywords:** guinea pig, feeding pattern, fecal pellet output, intestinal transit time, carmine red, gastroenterology

## Abstract

**Simple Summary:**

Guinea pigs are frequently used in gastrointestinal research, but knowledge on basic parameters connected with gastrointestinal physiological functions, including feed intake, fecal pellet output (FPO) and intestinal transit time, is incomplete. Recording such parameters in single- and pair-housed guinea pigs over 24 h revealed that they exhibit a diurnal feeding behavior, with peaks during the beginning and end of the light period, and FPO mirroring periods, with a defecation break during the night. In addition, we assessed transit time with a non-absorbable marker, counting approximately 5 h. Our findings can help to build a baseline data basis, important in the field of functional gastrointestinal animal physiology.

**Abstract:**

Guinea pigs are a traditional and frequently used species in gastrointestinal research. Comprehensive knowledge of basic parameters connected with their intestinal function, such as feed intake, fecal pellet output and gastrointestinal transit time, is important for evaluating results from basic gastrointestinal research that may be applied to practical problems in human and veterinary medicine, for example, when establishing diagnostic tools. Our study revealed that over a 24-h period, single-housed guinea pigs showed a continual but day-accentuated feeding activity, consuming 57% of the total feed during the light period, with pronounced peaks of feed intake during the beginning and end of the light period. This was mirrored by fecal pellet output during the light period and almost no defecation during the dark period, while potential coprophagy not measured in this study needs to be considered. A highly comparable feeding activity was recorded in pair-housed guinea pigs, with 60% of overall feed intake within the light period, indicating that such differences in housing conditions did not influence guinea pigs’ feeding behavior. Intestinal transit time was successfully recorded by oral administration of carmine red and counted 5 h on average. Hence, this study provides important information on the basic functional parameters of guinea pigs’ gastrointestinal tract physiology.

## 1. Introduction

Due to its gentle temperament, simple handling, low maintenance costs and intestinal tissue anatomy, the guinea pig is an important and traditionally used model in the field of gastrointestinal research including drug and vaccine development, studies on the enteric nervous system, studies on enteric infectious diseases and studies on the intestinal microbiome [1,2,3,4]. For proper evaluation of data including the suitability of a certain species to model the human intestinal physiology and pathophysiology [5], a preferably complete knowledge and understanding of basic physiological parameters connected with intestinal function(s) is crucial, bearing in mind that probably no single animal can perfectly mimic human conditions [6].

Additionally, with respect to the significant effect of periodic feeding behavior/energy intake on intermediary metabolism (for review, see [7]) and the concept of diurnality in intestinal motility [8], knowledge on basic data related to gastrointestinal functions is critical for proper evaluation of study results. This accounts for both basic research and, e.g., preclinical studies during drug development [9]. Data of interest connected with gastrointestinal physiological functions are, amongst others, feed intake (FI), fecal pellet output (FPO) and whole intestinal transit time (wITT). However, studies addressing such baseline data in guinea pigs are scarce. Two early studies exist, with one reporting a weak diurnal pattern in FI during a 24 h observation in guinea pigs [10]. The second one detected comparable meal sizes and intermeal intervals in young and adult guinea pigs, with a comparable distribution of meal numbers during a 24 h period [11]. In those studies, FI was recorded in group-housed animals, and hence data on a single-animal basis can only be extrapolated from the situations monitored. Studies assessing FPO in guinea pigs are most often performed in order to evaluate the effect of drugs or other factors modulating/affecting gastrointestinal motility, e.g., external stressors such as water avoidance stress [12,13,14,15,16,17]. However, to the best of our knowledge, no studies exist evaluating the periodicity of FPO in guinea pigs, especially related to their feeding behavior. Therefore, with the present study, we aimed to evaluate typical values of FI and FPO during daylight and nighttime in this species and to detect the potential effects of single- vs. pair-housing on these parameters in order to draw a more comprehensive picture of basic gastrointestinal functions. In addition, we measured wITT, using the non-absorbable marker carmine red, to compare our results with the already existing baseline data from studies using different techniques, such as intragastrical application of styrol particles or feeding radioactively labeled diets [18,19,20,21]. Using carmine red represents a method that is less stressful (no prior fasting is required) and less expensive (no complex measuring equipment needed) compared to the techniques in the cited studies.

## 2. Methods

### 2.1. Animals

Three different experiments were conducted to determine FI, FPO and wITT using a total of 30 adult Dunkin Hartley guinea pigs of both sexes, weighing 642 ± 207 g (10 to 12 weeks of age). Guinea pigs were bred and kept in the approved breeding and housing facility of the Institute for Physiology and Cell Biology at the University of Veterinary Medicine Hannover, Germany. Animals were housed in groups of 2 to 4 animals in cages with a solid cage base, with a size of 814 × 610 × 256 mm (L × W × H) and a floor area of 4000 cm^2^ (tecniplast GmbH, Hohenpeißenberg, Germany). Cages were equipped with wood shavings and one halved ~20 cm PVC tube per animal. Animals were kept under standardized conditions (20–24 °C room temperature, 60% humidity and a day/night cycle of 12:12 h, with 580 lux within the room animals were kept in, 30 lux within the cages during the light period and <10 lux during the dark period). Light turned on at 07:00 during experiments 1 and 3 and at 06:00 during experiment 2. Animals received a pelleted standard diet (g/kg DM: 168 crude protein, 31 crude fat, 130 crude fiber, 72 crude ash, 230 starch; 12.1 MJ ME, ssniff Spezialdiäten GmbH, Soest, Germany) and drinking water ad libitum. Fresh hay (20 g per animal) was provided at 07:00. This feeding regime was applied during all experiments. For recordings of FI at the single-animal level as well as of FPO and wITT, animals were separated 24 h prior to the start of the recordings and kept singly in cages, as described above, that were not their home cages, for proper adaptation. In those cases, cages were equipped with paper towel instead of wood shavings in order to enable easy fecal pellet collection. After finishing single animal recordings, animals were immediately returned to their home cages and former groups.

### 2.2. Experiment 1: 24-h Feed Intake and Fecal Pellet Output in Single-Housed Guinea Pigs

FI and FPO were initially determined in four single-housed guinea pigs (two male and two female) on one day for 24 h starting at 07:00. FI of pellets was determined by calculating the weight difference of the feed container at consecutive time points one hour apart. Feed containers were made of stainless steel with a 56 cm^2^ feed-filled area extending into the cage, allowing two animals to pick up feed simultaneously. They were easily detachable from the outside and equipped with a protection lid, ensuring minimal feed spillage by the guinea pigs. In addition, not yet consumed hay was collected and reweighed every hour for calculating consumed amount of hay, which was considered for calculation of overall FI. FPO was determined by collecting fecal pellets every hour, calculating the total number of expelled pellets and cumulative fecal pellet output (cFPO) defined as FPO during the first three hours after the start of collection. Disturbance of the animals was minimal as measurements and fecal pellet collection were finished within a few minutes and there was use of infrared light during the dark period.

### 2.3. Experiment 2: 24-h Feed Intake in Single-Housed and Pair-Housed Guinea Pigs

In order to determine whether diurnal variations in FI differed in single- compared to group-housed animals, FI was determined in six guinea pigs (four male, two female) under both conditions: animals were first kept singly housed, then returned to their home cages and reunited with their cage mates and, one week later, were kept pairwise during the measurements. FI measurements were conducted in the same manner as described for experiment 1. Due to the switch from summertime to wintertime prior to the experiment, lights were now turned on from 06:00 to 18:00.

### 2.4. Experiment 3: Determination of Whole Intestinal Transit Time

wITT was determined in 10 single-housed male and 10 female, non-fasted animals on a total of 13 different experimental days (1 to 4 animals each experimental day) starting at 07:00 and ending at 17:00 by oral application of the non-absorbable marker carmine red and checking pellets for red coloring every 15 min. Each guinea pig was orally administered a 6% carmine red solution (Sigma-Aldrich C1022) dissolved in 0.5% methylcellulose (Sigma-Aldrich 274429, 0.5 mL/100 g bodyweight (BW)) a single time at 07:00. Preliminary experiments administering different amounts of the 6% carmine red solution revealed that the chosen solution and amount were sufficient for intensive coloring of fecal pellets (see Supplementary Materials Figure S1), without any side effects for the animal. wITT was defined as the time of appearance of the first red colored fecal pellet, and guinea pigs were further observed for their well-being until 17:00. Afterwards, they were returned to their home cage. In addition to wITT, FI and FPO were measured in these animals between 07:00 and 17:00 as described above.

### 2.5. Statistics

Recorded FI was calculated based on BW of the individual animal. All data are presented as means ± standard deviation (SD). FI data of single-housed and pair-housed animals were analyzed by two-way ANOVA with subject/animal and different times of the day representing the factors, and by Sidak’s multiple comparison test to test for differences between single time points and groups. FI data obtained in experiment 3 were analyzed by two-way ANOVA with sex and different times of the day as factors for evaluation of sex-specific differences. Comparison of total FI and FPO in single-housed animals in experiment 1 during the 24-h period and during the 12-h light and dark period was conducted by the paired *t*-test. Comparison of total FI during the 24-h period and during the 12 h light and dark period in single-housed animals in experiment 1 plus 2 and pair-housed animals in experiment 2 was conducted by the unpaired *t*-test. Hourly FI recorded in single-housed animals in experiment 2 was correlated with corresponding values of the same animals housed pairwise, and the Spearman correlation coefficient was computed. Comparison of wITT in male and female animals in experiment 3 was conducted by the unpaired *t*-test. All statistical analysis was performed using GraphPad Prism 9.00 (GraphPad Software Inc., La Jolla, CA, USA). All *p* values < 0.05 were considered as statistically significant. Asterisks indicate significance. Statistical tests were applied as indicated in figure legends. *N* numbers indicated in figure legends represent numbers of animals.

## 3. Results

### 3.1. Experiment 1: 24-h Feed Intake and Fecal Pellet Output in Single-Housed Guinea Pigs

**Figure 1:**
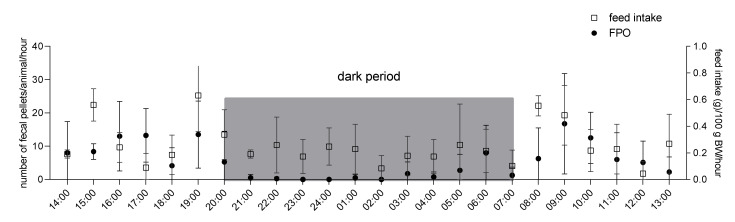
Fecal pellet output (FPO) aligned with feed intake (FI) in single-housed guinea pigs during 24-h recording period. *n* = 4; means ± SD. Times on the *x* axis indicate end time for each hour (e.g., 14:00: values measured between 13:00 and 14:00). Dark gray box indicates values measured during the dark period.

Figure 1 shows FI and FPO during 24-h recordings from 07:00 to 07:00 in single-housed guinea pigs. In Supplementary Materials Figure S2, individual patterns of FI and FPO for each of the four animals are depicted. Guinea pigs showed a consistent diurnal variation in FI with three periods of higher feeding activity: one at the beginning; a second, weaker one after two thirds of the light period; and one at the end of the light period. Their FI was lowest during midday (12:00). During the dark period, the animals showed the highest FI right after the lights turned off (20:00), followed by a consistent moderate FI with no clear peak. Results of two-way ANOVA revealed highly significant variation in FI between hours (*p* < 0.001) and a statistical trend towards variation in FI of different guinea pigs (*p* = 0.07; data not shown). Comparing the total amount of feed consumed during the light and the dark period, a significant higher FI during the light period was revealed (3.69 ± 0.68 g/100g BW vs. 2.45 ±0.57 g/100 g BW, *p* < 0.01, data not shown). FPO peaked in the morning and evening between 08:00 and 09:00 and 18:00 and 19:00, respectively, was also high between 15:00 and 17:00 and hence was associated with a prior high FI. Overall FPO was much higher during the light period than during the dark period: on average, animals expelled 109.13 ± 17.2 pellets during the light period vs. 21.0 ± 13.11 pellets during the dark period (*p* < 0.001; data not shown). During the dark period, almost no fecal pellets could be collected between 20:00 and 05:00. Two-way analysis of variance revealed significant differences in the number of fecal pellets expelled at different times of the day (*p* < 0.01) but no statistically significant variation based on the animal (*p* = 0.55). However, cFPO varied greatly between individual animals from 6 to 74 pellets during the first three hours of collection and was 35.5 ± 29.4 pellets on average (data not shown).

### 3.2. Experiment 2: 24-h Feed Intake in Single-Housed and Pair-Housed Guinea Pigs

**Figure 2:**
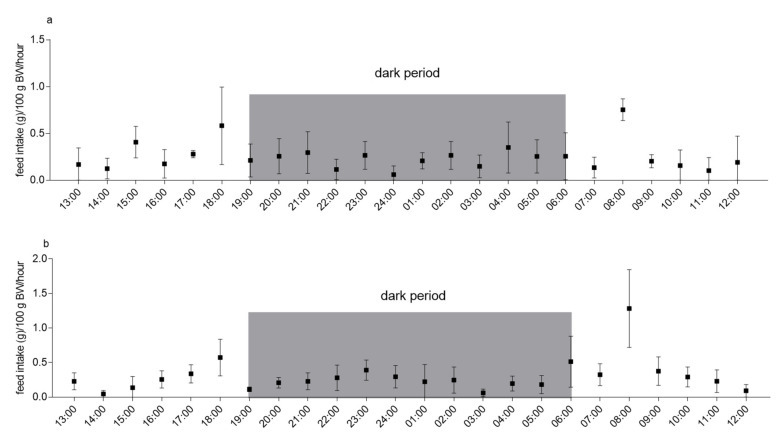
Feed intake (FI) in guinea pigs during 24 h recording period under single-housing (**a**) and pair-housing (**b**) conditions. *n* = 6; means ± SD. Times on the x axis indicate end time for each hour (e.g., 14:00: values measured between 13:00 and 14:00). Dark gray boxes indicate values measured during the dark period.

Figure 2 shows FI under single-housing (a) and pair-housing (b) conditions in six animals in a paired experimental study design. Under both housing conditions, the main feeding activity was recorded at the beginning (07:00 to 08:00) and the end (17:00 to 18:00) of the light period. In addition, single-housed animals showed an increased FI between 14:00 and 15:00, while pair-housed animals increased their FI in a more continuous manner, starting from 15:00 until the peak between 17:00 and 18:00. However, values between 14:00 and 15:00 were not significantly different (*p* = 0.13). When housed pairwise, guinea pigs additionally showed increased FI close to the middle of the dark period (22:00 to 23:00) and one hour before the lights turned on (05:00 to 06:00). This was not detectable under single-housing conditions and did not show any statistical significance (*p* = 0.89 and *p* = 0.18, respectively). Two-way ANOVA revealed a highly significant variation in FI at different times under both conditions (*p* < 0.001) and a trend for significantly different FIs of different guinea pigs when single-housed (*p* = 0.05), which reached statistical significance under pair-housing conditions (*p* < 0.001; data not shown). However, values recorded from single-housed animals significantly correlated with values from the same animals when pair-housed (*p* < 0.01, Spearman r = 0.25), indicating that guinea pigs did not exhibit great variances in their diurnal feeding pattern irrespective of housing conditions (Supplementary Materials Figure S3).

**Figure 3:**
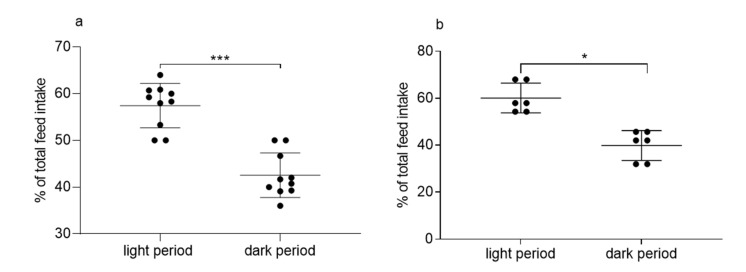
Comparison of feed intake (FI) in single-housed (**a**) and pair-housed (**b**) guinea pigs during light and dark periods. *n* = 10 (panel a) and *n* = 6 (panel b); means ± SD. Paired *t*-test, * *p* < 0.05, *** *p* < 0.001.

Additionally, when considering single-housed animals from experiments 1 and 2 for calculation of total FI during 24 h, values were comparable in single-housed (6.04 ± 1.2 g/100 g BW) and pair-housed (7.14 ± 2.46 g/100 g BW) guinea pigs (*p* = 0.29, Supplementary Materials Figure S4). Comparing FI between single-housed and pair-housed animals separately during the light and the dark period revealed no significant differences between the housing groups (light period: 3.45 ± 0.64 g/100g BW and 4.19 ± 1.03 g/100g BW, respectively; *p* = 0.09; dark period: 2.59 ± 0.69 g/100g BW and 2.49 ± 1.21 g/100 g BW, respectively; *p* = 0.46). These values corresponded to 57% of FI during the light period in single-housed animals, which was significantly more than during the dark period (Figure 3a). Comparable values were computed for pair-housed animals in experiment 2 (60% FI during the light period, Figure 3b).

### 3.3. Experiment 3: Whole Intestinal Transit Time

On average, the first red-colored fecal pellet appeared 4.8 ± 2.12 h after oral application of carmine red, with a minimum of 2.45 h and a maximum of 8 h for single animals (Supplementary Materials Figure S4). No differences between sexes were detected (*p* = 0.15, data not shown). Within the observation period (07:00 to 17:00), animals showed the highest FI between 07:00 and 08:00 in the morning and the lowest FI around midday (11:00 to 12:00). FPO peaked one hour after the highest FI between 08:00 and 09:00 (Supplementary Materials Figure S4). Two-way ANOVA revealed no significant differences in FI between sexes (*p* = 0.83; Supplementary Materials Figure S5).

## 4. Discussion

In contrast to mice and rats, data on basic parameters reflecting intestinal functions such as FI, FPO and wITT, especially in a combined and comprehensive manner, are missing for the guinea pig, thus far. As FI strongly influences intestinal functions such as motility and secretion [22] and, on the other hand, can be affected by many internal and external factors, monitoring of the free-feeding behavior in non-treated animals, but under a standardized, laboratory setting, can create the basis for further experiments [23]. An early study already showed a sex-independent, diurnal fluctuation in FI in group-housed guinea pigs kept on a 12:12 h light/dark schedule with a period of increased FI at the beginning and end of the light period and in the middle of the dark period [10]. We recorded a comparable feeding pattern with peaks at the beginning and the end of the light period in both single- and pair-housed guinea pigs and an additional increased feeding activity between 14:00 and 15:00 in single-housed animals, as well as a more continuously increasing FI starting from 15:00 in pair-housed animals. This pattern of increased FI after two thirds of the light period is also well comparable to Horton’s results [10]. However, a clear peak close to the middle of the dark period was only detectable in pair-housed guinea pigs. Single-housed animals showed an overall less regular course of FI during the dark period without a clearly detectable peak, as also reflected by the computed weak correlation coefficient. The effect of housing conditions on feeding behavior has also been described for rats: they ate more quickly and spent overall less time per day eating when group-housed compared to when they were housed singly, which was discussed to be induced by higher interaction with group mates (and hence less time for eating) and by not always having access to the food cup at their preferred eating time [24]. However, in contrast to the latter study and studies in mice [25] and rabbits [26] showing either higher or lower overall FI in group-housed animals, neither hourly FI nor total FI during 24 h was significantly different in single- and pair-housed guinea pigs in our study. This may be due to species-specific differences regarding their daily activity pattern (diurnal/crepuscular guinea pigs vs. nocturnal mice) and length of the study (e.g., mice were studied for 18 months) and influenced by the great individual differences in FI as seen for the animals in our study and for rabbits in Horton’s study [27]. Interestingly, single-housed guinea pigs in experiment 2 showed peaks (despite in the morning when hay was provided) and lows in FI exactly one hour earlier than guinea pigs in experiment 1 when the lights turned on and off one hour later than in experiment 2. This ability to adapt feeding behavior to changes in the lighting cycle has already been reported by Horton for guinea pigs [10] and rabbits [27]. We recorded approximately 57 to 60% of overall FI during the light period and 43 to 40% during the dark period for single-housed and pair-housed guinea pigs, respectively. This is well comparable to the values reported in the study by Horton with 52 to 55% of total feed consumed during the day [10]. Despite the recorded higher amount of feed consumed during the light period in our study, the recorded 24-h FI pattern shows that guinea pigs obviously do not exhibit a period without any FI during the dark period, fitting to the results of Hirsch that show a comparable number of meals during light and dark periods in this species [11]. Our results can be related to data from studies on domesticated and laboratory guinea pigs, showing that this species is more or less continuously active throughout a 12:12 h light/dark cycle but exhibits the greatest physical activity in the early morning and evening [28,29,30,31,32,33] and hence is best assignable to a crepuscular species. This assumption is further supported by our results of FPO from experiment 1, which mirrored a higher FI during the light period and a very low feces deposition during most of the time in the dark period. The 09:00 peak of FPO approximately one hour after the highest FI during the light period indicates gastrocolic reflex in response to feed ingestion, which is known to be most active during the morning time and immediately after meals [34] and could also be detected at 16:00 and 17:00 following increased feeding activity at 15:00. Fewer feces deposition in the non- or low-active phase has also been shown for the clearly nocturnal rat [35] and for the diurnal degu [36]. The degu is especially interesting in comparison to the guinea pig because both species exhibit coprophagy. For the diurnal degu, it was shown that a higher number of fecal pellets were deposited during the day than during the night, and this was influenced by a significantly greater nocturnal coprophagy [36]. We did not record coprophagy in our guinea pigs, which represents one weakness of the present study and should be explored in further experiments on the feeding behavior of guinea pigs. However, it was reported that 70 to 80% of the total coprophagy rate in the guinea pig occurred during the light phase [37]. Based on that, the significantly lower fecal pellet discharge during the dark period in our study indicates the night as the period of lower digestive and probably lower overall activity in guinea pigs. The FPO assay as a validated, non-invasive tool for (distal) colonic motility assessment [38] has been frequently used in mice and rats [35,39,40,41,42,43,44] and also in guinea pigs in early preclinical drug development studies to predict effects of particular compounds on gastrointestinal motility [12,13,14,15,16]. The recorded 3-h cFPO in our study exceeded recorded values in control animals from the above-cited studies, considering that Hussain’s study from 2017 was the only one evaluating 3-h cFPO, while in the other studies, 4-h or 6-h cFPO was recorded. However, the 3-h cFPO of 7.8 ± 4.4 and the 6-h cFPO of 15.22 ± 15.78 pellets recorded by Hussain and colleagues [12,14], as well as the 4-h cFPO of approximately 15 pellets recorded by Park [16], were markedly smaller than ours. One explanation might be differences in the diets provided, since our animals received and consumed hay, which has been shown to lead to greater FPO in rabbits [45]. In addition, intragastric or intraperitoneal vehicle injection or food deprivation overnight as applied in Park’s study might have affected the FPO of the animals in those studies. Furthermore, in the mentioned studies, no information is provided on the time of day the cFPO measurements were performed, which might have been different from ours and could explain the recorded differences. Our data on the 24 h distribution of FPO in guinea pigs and the great inter-individual differences in the cFPO highlight the importance of the time of the day chosen for those measurements and the physiological differences between individuals, which was also highlighted for rats [35].

There are several methods for measuring intestinal transit time in animals, including oral administration of non-absorbable markers, which has frequently been conducted in rats and mice [35,46,47,48,49,50,51]. We selected the use of orally dosed carmine red as a color marker. It is cheap (no complex measuring equipment is needed), easy to use and causes little stress (does not require prior fasting). Studies using this marker for measuring intestinal transit time in guinea pigs and its modulations by specific drugs introduced the dye via a colonic cannula and hence specifically measured colonic transit time, which was between 220 and 310 min [52,53]. Since the experimental procedure in those studies was quite different from ours and animals assigned to the control group were subcutaneously administered a vehicle compound, the reported values do not represent baseline data comparable to ours. The average transit time we obtained was consistent with the range of values (typically 3–5 h) seen in studies with Co-EDTA [20,21] or chromium dioxide administered in the diet [19] and intragastrically administered 300 to 500 µm-sized styrol particles [18].

In studies focusing on gastric emptying and small or large ITT in guinea pigs as affected by different drugs, ^51^Cr as a radiomarker and charcoal were used, and transit times were presented as the percentage of the distribution or migration of the marker within the defined intestinal segments [16,17,54].

## 5. Conclusions

The results of the present study provide baseline physiological data on intestinal functions in the guinea pig. This study highlights the fact that this species, kept under laboratory conditions, (1) exhibits a crepuscular pattern of FI with distinct peaks during the light period and overall higher FI during the light than the dark period, which is (2) more or less unaffected by housing conditions (single- vs. pair-housing), (3) shows a clear diurnal pattern of FPO with almost no FPO during the night and great individual differences regarding cFPO during the first 3 h of collection and (4) has a wITT of approximately 5 h determined by oral administration of carmine red. Since guinea pigs are very social, they should be at least housed in pairs whenever possible, also when kept for scientific purposes. From our study results, we can conclude that FI is not dramatically affected by housing conditions and that separation of animals is possible if necessary, for specific measurements, when adequate adaptation is provided. With regard to our results of (c)FPO, researchers should consider the time of the day those measurements are conducted and the diet provided and provide information on those when publishing such data.

In future studies, more animals should be included to determine potential age- and sex-specific differences under distinct housing conditions and to estimate to which extent nocturnal coprophagy contributes to the recorded low overall FPO during the dark period. To further evaluate to which extent the guinea pig’s intestines exhibit diurnal patterns with respect to loading and unloading, in vivo intraluminal motility and pressure measurements using, e.g., wireless motility capsules could be considered [9].

## Data Availability

The data presented in this study are available on request from the corresponding author.

## Supplementary Materials

The following are available online at https://www.mdpi.com/article/10.3390/ani11061593/s1, Figure S1: “Preliminary testing of different amounts of orally administered 6% carmine red solution leading to coloring of fecal pellets. Pictures show the first red pellet detected by visual inspection”, Figure S2: “Fecal pellet output (FPO) aligned to feed intake (FI) of each of the four single-housed guinea pigs in experiment 1”. Figure S3: “Correlation of hourly feed intake (FI) of guinea pigs”. Figure S4: “Fecal pellet output (FPO) aligned to feed intake (FI) in single-housed guinea pigs in experiment 3 between 07:00 and 17:00 during the light period.”. Figure S5: “Feed intake (FI) in male and female guinea pigs between 07:00 and 17:00 during the light period in experiment 3”.
