# Supplementary material for: Good to Know: Baseline Data on Feed Intake, Fecal Pellet Output and Intestinal Transit Time in Guinea Pig as a Frequently Used Model in Gastrointestinal Research
Source: Animals (Basel). 2021 May 28;11(6):1593. doi: 10.3390/ani11061593 (PMC8227794; doi:10.3390/ani11061593)
Supplement: Supplementary file 1 [file animals-11-01593-s001.zip › animals-1185912-supplementary/Supplementary figure after proof.pdf]

**Supplementary figures**

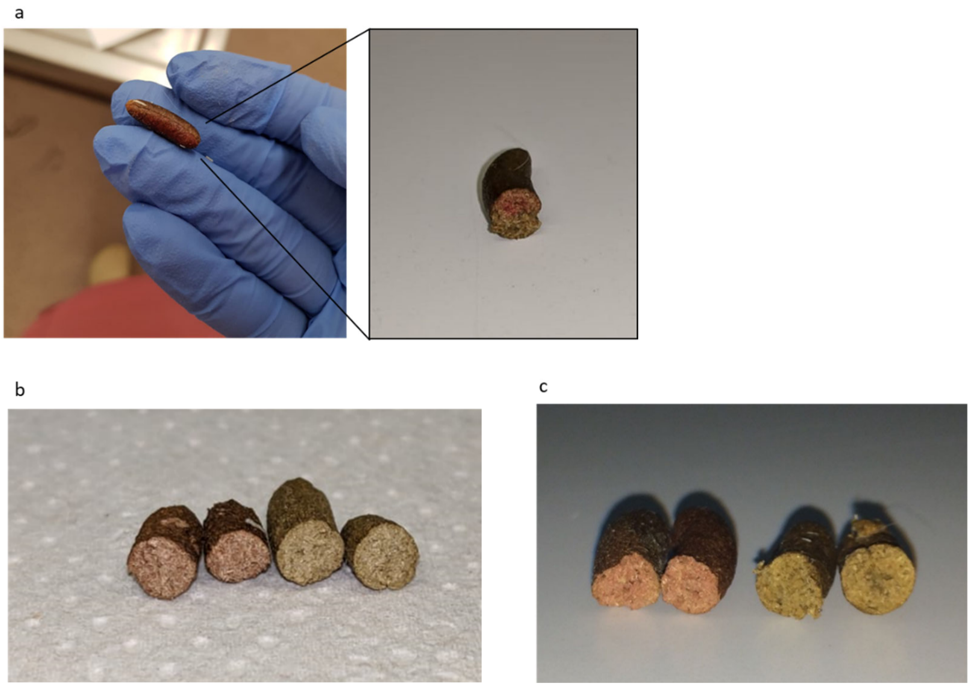


**Supplementary figure S1**: preliminary testing of different amounts of orally administered 6% carmine red solution leading to coloring of fecal pellets. Pictures show the first red pellet detected by visual inspection. (**a**) First red fecal pellet after oral administration of 0.25 ml/100 g BW 6% carmine red. Note, only faint and incomplete staining of the pellet outside and inside (displayed panel a). (**b**) First red fecal pellet after oral administration of 0.50 ml / 100 g BW 6% carmine red. (**c**) First red fecal pellet after oral administration of 1.0 ml / 100 g BW 6% carmine red

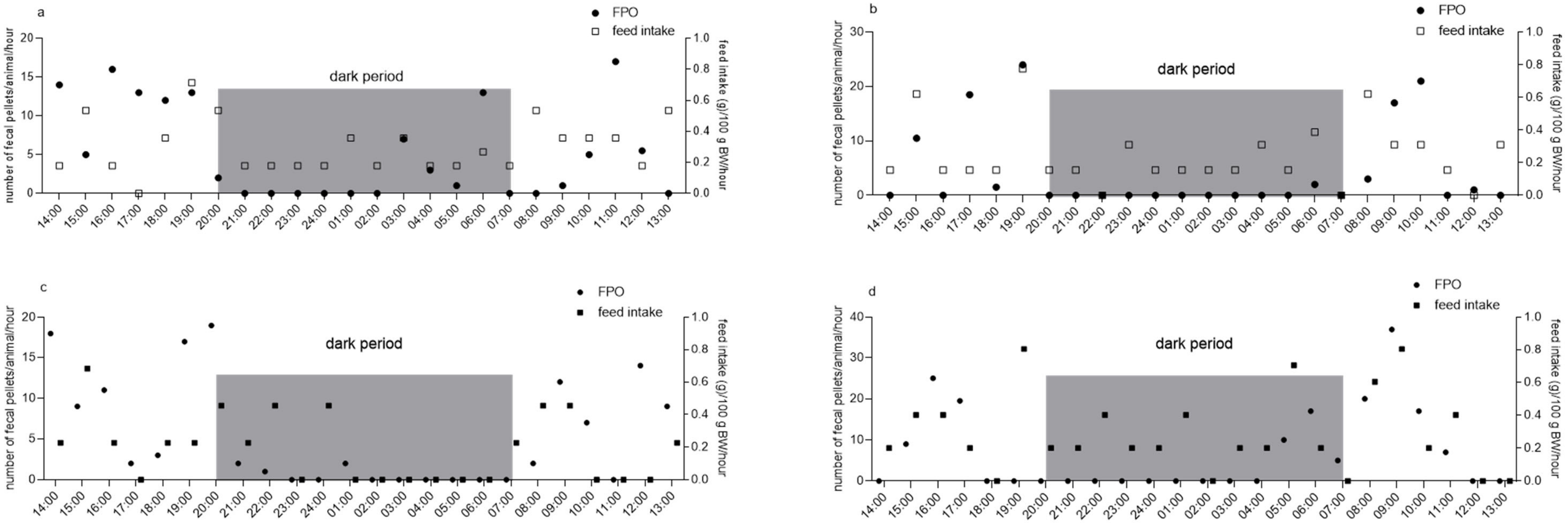


**Figure S2**: fecal pellet output (FPO) aligned to feed intake (FI) of each of the four single-housed guinea pigs in experiment 1; means ± SD. Times on the x axis indicate end time for each hour (e.g. 14:00: values measured between 13:00 and 14:00). Dark grey box indicates values measured during the dark period (07:00 to 19:00). Each panel (**a–d**) represents values recorded from one animal.

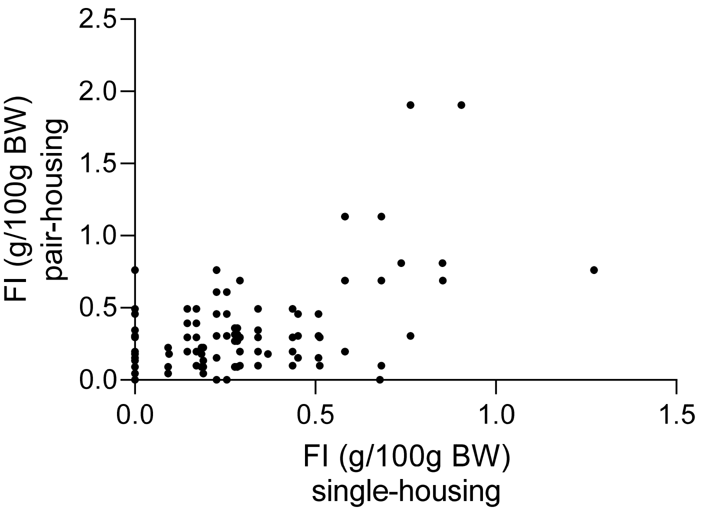


**Figure S3**: correlation of hourly feed intake (FI) of guinea pigs under single-housing and pair-housing conditions during a 24 h recording period in experiment 2. $n = 6$, 144 pairs; Spearman $r = 0.25$, $p < 0.01$.

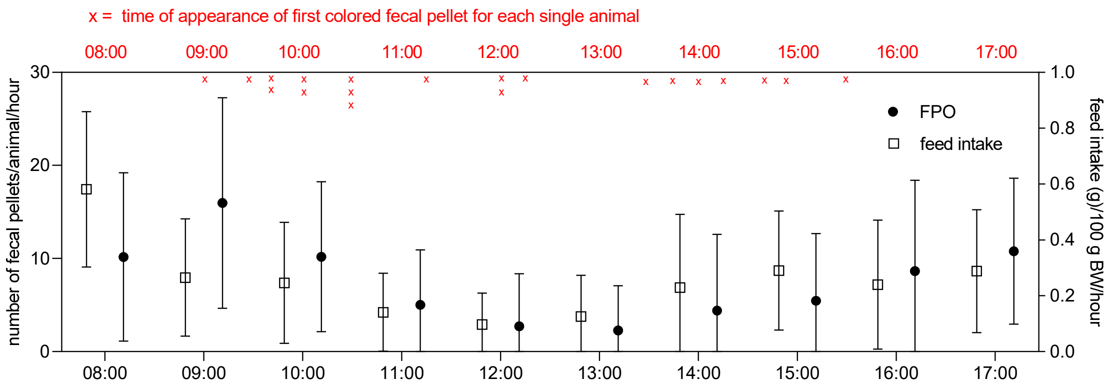


**Figure S4**: fecal pellet output (FPO) aligned to feed intake (FI) in single-housed guinea pigs in experiment 3 between 07:00 and 17:00 during the light period. Times on the x axis indicate end time for each hour (e.g. 14:00: values measured between 13:00 and 14:00). Animals received a 6% carmine red solution (0.5 ml / 100 g bodyweight) for determination of intestinal transit time at 07:00. Red crosses indicate time point of appearance of first red colored fecal pellet for individual animals. *n* = 20; means ± SD.

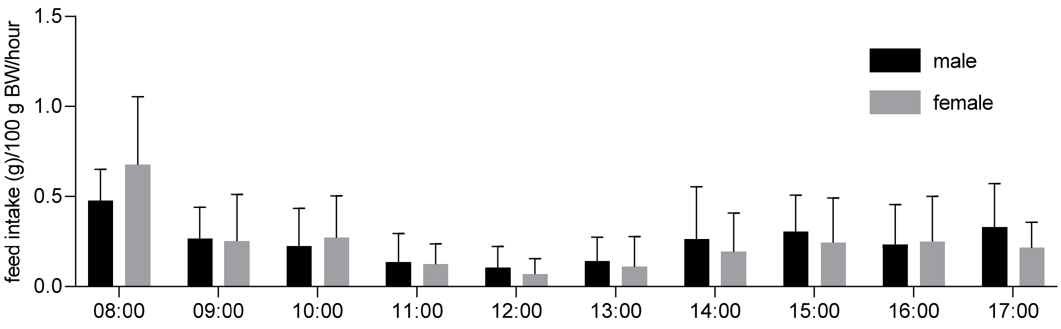


**Figure S5**: feed intake (FI) in male and female guinea pigs between 07:00 and 17:00 during the light period in experiment 3; times on the x axis indicate end time for each hour (e.g. 14:00: values measured between 13:00 and 14:00). two-way-ANOVA revealed no differences between sexes and a significant impact by time of the day ($p < 0.001$). $n = 20$ (10 male, 10 female); means ± SD.
